# Bridging the Language Gap: The Role of Human-Mediated Translation in Japanese Medical Settings

**DOI:** 10.7759/cureus.50493

**Published:** 2023-12-14

**Authors:** Yudai Kaneda, Morihito Takita, Makoto Kosaka, Tamae Hamaki, Kazutaka Hosoda, Eiji Kusumi, Masahiro Kami, Tetsuya Tanimoto

**Affiliations:** 1 Medicine, Hokkaido University, Sapporo, JPN; 2 Internal Medicine, Medical Governance Research Institute, Tokyo, JPN; 3 Medicine, Imamura General Hospital, Kagoshima, JPN; 4 Internal Medicine, Navitas Clinic Shinjuku, Tokyo, JPN; 5 Internal Medicine, Accessible Rail Medical Services Tetsuikai, Navitas Clinic Shinjuku, Tokyo, JPN; 6 Internal Medicine, Accessible Rail Medical Services Tetsuikai, Navitas Clinic Tachikawa, Tachikawa, JPN; 7 Internal Medicine, Accessible Rail Medical Services Tetsuikai, Navitas Clinic Kawasaki, Kanagawa, JPN

**Keywords:** japanese medical settings, communication, foreign residents, language barrier, human-mediated translation

## Abstract

Introduction

Foreign residents in Japan often face challenges accessing healthcare due to language barriers, potentially leading to health inequities. This study aimed to assess the utilization and impact of human-mediated translation services in a specific medical setting in Tokyo.

Methods

A retrospective investigation was conducted on medical records of foreign patients who utilized human-mediated translation services at Navitas Clinic Tachikawa (Tachikawa, Tokyo, Japan) from November 2017 to December 2021. Data on age, gender, language used, department visited, diagnosis, insurance status, and booking methods were analyzed.

Results

Out of the 124 foreign patients who utilized the human-mediated translation services during the study period, 69 (56%) were male, and 55 (44%) were female. The median age was 35 years, with a range from 3 to 61 years. English was the predominant language used by 34 patients (59%), followed by Chinese for nine patients (16%) and Spanish for four patients (7%). The majority, 107 patients (86%) visited the internal medicine department, nine patients (7%) consulted dermatology, and six patients (5%) visited pediatrics. Regarding insurance status, 47 patients (81%) were insured, three patients (5%) were uninsured by the Japanese national health insurance system, and eight patients (14%) were self-pay. The primary mode of appointment booking was at the reception desk, with 112 patients (90%) using this method, while 12 patients (10%) made reservations online.

Conclusions

The findings of this study underscore the importance of human-mediated translation services for improving healthcare accessibility for foreign residents in Japan, emphasizing the need to address language barriers and promote health equity in clinical settings. Future studies should also explore challenges faced in patient-physician interactions from a linguistic perspective and potential technological solutions to enhance these services.

## Introduction

The medical challenges faced by foreigners or immigrants in accessing healthcare in foreign countries, which result in health disparities and deterioration in health conditions, can be viewed as universal health inequities [1-3]. Indeed, foreign residents may need help utilizing medical services due to their unfamiliarity with the healthcare system [3]. In addition, these foreign residents may not be enrolled in health insurance or need more awareness of the available options [4]. The language barrier is also a particularly significant issue for foreign residents seeking to receive medical care in Japan. Communication difficulties can lead to anxiety and inconvenience, potentially making it challenging for healthcare professionals to understand the needs of their patients [5].

In this context, foreign residents could be a group heavily impacted by the novel coronavirus disease 2019 (COVID-19). The COVID-19 pandemic has been a significant factor causing substantial disruption to healthcare services globally. Particularly in Japan, the pandemic has led to social distancing and fear of transmission, causing a decrease in hospital visits and reports of individuals ignoring symptoms until their conditions worsen [6,7]. Therefore, healthcare professionals must understand foreign residents' communication needs and strive to provide language support as necessary [5].

In order to provide linguistic support for physicians in Japanese medical settings, a telephone-based human interpretation service is available. This service, called MediPhone, developed by a Japanese medical SaaS startup founded by former tech industry professionals, offers remote medical interpretation over the phone [8]. It acts as a bridge, connecting healthcare professionals with remote medical interpreters in real time, thereby facilitating communication between healthcare professionals and foreign patients who speak different languages. The service covers 31 languages and boasts a roster of over 300 registered medical interpreters. Users can access the service for free for up to 30 minutes per month, with additional charges incurred for extended usage. A distinct advantage of this platform is the employment of human interpreters. If there is any ambiguity in the patient's statements, the interpreter can engage in a clarifying dialogue with the patient before relaying the information to the medical staff, ensuring the patient's narrative is accurately conveyed.

In previous studies, health inequity has been explored through four primary aspects: 1) race/ethnicity, 2) primary language, 3) gender, and 4) location [1]. Especially concerning primary language, Japanese is recognized as one of the most challenging languages, and the English proficiency of many Japanese medical professionals remains relatively low [5,9]. Given this context, human-mediated translation services in clinical practice are of paramount importance. However, it remains uncertain which specific patient groups most require these interpreter services and the circumstances driving this need. This study, therefore, was designed to highlight the patients who may face challenges accessing medical care due to language barriers in a single medical clinic.

## Materials and methods

Study site

The study site was in Tokyo, where the number of foreign residents was reported to be approximately 540,000, accounting for about 20% of the foreign population living in Japan in 2022 [10]. Particularly in Tachikawa City, which has a population of over 180,000, there are around 4,650 foreign residents, and remarkably, despite the challenges of the pandemic, their numbers have been on the rise for the past seven years, indicating a latent demand for medical care tailored to foreigners [11]. Its strategic location and service offerings make it an ideal setting for examining healthcare service utilization among foreign residents in a metropolitan area.

Settings and participants

Navitas Clinic Tachikawa is an outpatient clinic (Tachikawa, Tokyo, Japan), consisting of internal medicine, pediatrics, and dermatology departments. The clinic also offers vaccinations and health check-ups. The clinic operates until 9 p.m. on weekdays and until 5 p.m. on Saturdays, providing a service format accessible to those working in central Tokyo who typically find it challenging to visit clinics during the daytime. The clinic is staffed with seven physicians, two pediatricians, and three dermatologists, all of whom are Japanese and speak Japanese as their first language. The clinic started implementing human-mediated translation services in November 2017. The target group for this study comprised foreign patients who utilized human-mediated translation services for consultations at our clinic between November 2017 and December 2021. This period was selected to capture a comprehensive dataset post-implementation of the translation services.

Data collection and analysis

We conducted a retrospective investigation of the medical records of foreign patients who used human-mediated translation services for consultations at our clinic from November 2017 to December 2021. The surveyed items included age, gender, the language used, department visited, diagnosis, whether any tests were conducted, whether health insurance was in place, methods of booking, and dates of visits. These variables were selected to provide a comprehensive overview of patient demographics and their interaction with the healthcare system. To ensure accuracy and comprehensiveness, data extraction was performed by a trained team of medical staff under strict confidentiality protocols. This approach aimed to identify patterns in service utilization and pinpoint specific needs of foreign residents within the healthcare system.

## Results

Table 1 presents the results of the visit list. During the study period, a total of 124 foreign patients had consultations using human-mediated translation services. Of these, 69 (56%) were male, and 55 (44%) were female. The median age was 35 years, ranging from 3 to 61 years. By age group, there were six patients (5%) aged 10 and under, no patients (0%) in their teens, 27 patients (22%) in their 20s, 82 patients (66%) in their 30s, five patients (4%) in their 40s, two patients (2%) in their 50s, and two patients (2%) in their 60s. By department, 107 patients (86%) visited internal medicine, nine patients (7%) visited dermatology, and six patients (5%) visited pediatrics. Two patients (2%) underwent COVID-19 antibody testing. In terms of reservation format, 12 patients (10%) made online reservations, and 112 patients (90%) registered at the reception desk.

**Table 1:** Clinical characteristics of visiting patients.

	Number (%) (N=124)
Age	
0–9	6 (5)
10–19	0 (0)
20–29	27 (22)
30–39	82 (66)
40–49	5 (4)
50–59	2 (2)
60–69	2 (2)
Age (median, interquartile range [IQR])	35 (29-38)
Sex	
Male	69 (56)
Female	55 (44)
Department	
Internal medicine	107 (86)
Dermatology	9 (98)
Pediatrics	6 (5)
COVID-19 antibody test	2 (2)
Methods of booking	
WEB	12 (10)
Reception	112 (90)

Of these, 30 males (52%) and 28 females (48%), a total of 58, were registered on the patient list. The characteristics of these individuals are summarized in Table 2. The median age was 34 years. The languages used on human-mediated translation services were English for 34 patients (59%); Chinese for nine (16%); Spanish for four (7%); Hindi for three (5%); Nepalese for two (3%); and Russian, Thai, Vietnamese, French, Tagalog, and Bengali each for one patient (1%). Forty-seven patients (81%) were covered by the Japanese National Insurance, three patients (5%) did not have insurance, and eight patients (14%) were self-pay. Of the three patients without insurance, two were tourists, and one had forgotten to renew. There were eight patients (14%) who only had tests, 23 patients (40%) who had both tests and prescriptions; 22 patients (38%) who only had prescriptions; one patient (1%) who had a test, a prescription, and a referral; two patients (3%) who only had a consultation; one patient (1%) who only had a referral; and one patient (1%) who had a referral and was hospitalized. The most common condition was the common cold, affecting 29 patients (50%), and pre-return COVID-19 tests for border entry screening were performed on five patients (9%). There were also two patients (3%) each for HPV vaccination and hay fever, and one patient each (1%) for diabetes, acute gastritis, and urinary tract infection.

**Table 2:** Characteristics of the patient list.

	Number (%) (N=58)
Age (median, interquartile range (IQR))	34 (28-38)
Sex	
Male	30 (52)
Female	28 (48)
Language	
English	34 (59)
Chinese	9 (16)
Spanish	4 (7)
Hindu	3 (5)
Nepalese	2 (3)
Others	6 (10)
Insurance	
Yes	47 (81)
No	3 (5)
Out-of-pocket expense	8 (14)
Test	
Examination	8 (14)
Examination + Prescription	23 (40)
Prescription	22 (38)
Consultation	2 (3)
Main symptom	
Common cold	29 (50)
Pre-return COVID-19 tests	5 (9)
HPV vaccination	2 (3)
Hay fever	2 (3)
Others	20 (34)

## Discussion

The results of this study demonstrate a clear need for human-mediated translation services, particularly for foreign residents visiting Navitas Clinic Tachikawa in Tokyo. During the study period, 124 foreign patients utilized human-mediated translation services, indicating a significant demand for language interpretation services in healthcare settings. The gender distribution was relatively balanced, and the median age of patients was 35 years. Regarding language needs, English was the most commonly used language in human-mediated translation services, followed by Chinese and Spanish.

Notably, a large proportion of these patients were in their 30s, possibly reflecting the age distribution of foreign workers, with an average age reported to be 33.3 in Japan [12]. This information is especially useful for medical institutions planning resources and services for foreigners, such as in Osaka and Aichi, where there are large numbers of foreign workers [13]. In addition, 86% of the patients who visited our clinic had an internal medicine consultation, suggesting that they had a chronic disease or general health condition that required regular visits [14].

The fact that most patients had health insurance, and the majority of patients booked their appointments at the reception desk, might be indicative of a certain level of familiarity with the Japanese healthcare system among foreign residents. However, the three patients who did not have insurance highlight an important issue. Insurance enrollment is a key factor in ensuring access to healthcare services [15]. Healthcare professionals and policymakers need to address this issue to prevent health disparities among foreign residents.

This study has some limitations. Firstly, human-mediated translation services are not always the go-to solution for straightforward English conversations. Other general digital translation tools, such as Google Translate and DeepL, or even non-verbal communication can influence the preference for or against using human-mediated translation services. Translation services might not be required for brief medical examinations conducted by physicians fluent in English. In addition, since some patients speak languages not covered by human-mediated translation services, or are seen with friends who speak Japanese, caution should be taken in interpreting this survey. Secondly, being a single-centre study, the findings may not fully represent all medical clinics in Japan. Thirdly, the study did not collect information about the patient's nationality, length of stay in Japan, or level of Japanese proficiency, which could influence the need for interpretation services. Consequently, future research should validate the efficacy of such human-mediated translation services by comparing consultation durations and the accuracy of comprehension.

Despite these limitations, our study provides valuable insights into the utilization of medical interpretation services by foreign residents in Japan. Given the increasing globalization and diversity in society, the demand for such services will likely continue to grow. Human-mediated translation services play a crucial role in ensuring that foreign residents have equal access to healthcare services, ultimately contributing to the goal of health equity.

## Conclusions

In conclusion, this study underscores the vital importance of human-mediated translation services in enhancing healthcare accessibility for foreign residents in Japan, emphasizing their diverse linguistic needs and varied medical services they utilize. Future research should focus on expanding the scope of these studies to include multiple centers and diverse patient demographics, further exploring the impact of translation services on patient care quality and healthcare accessibility for foreign residents in Japan. This study, thus, serves as a foundational step in understanding and addressing the unique healthcare needs of Japan's increasingly diverse population.
